# Indicators of exposure to physical violence among adolescents, Pernambuco, 2006-2022: time series

**DOI:** 10.1590/S2237-96222026v35e20250215.en

**Published:** 2026-05-13

**Authors:** Cláudia Fabiane Gomes Gonçalves, Mauro Virgílio Gomes Barros, Lygia Maria Pereira da Silva, Marco Aurélio de Valois Correia Junior, Emilia Chagas Costa, Caroline Ramos de Moura Silva

**Affiliations:** 1 Universidade de Pernambuco, Programa de Pós-graduação em Hebiatria, Recife, PE, Brazil Universidade de Pernambuco Programa de Pós-graduação em Hebiatria Recife PE Brazil; 2 Universidade de Pernambuco, Programa de Pós-graduação em Educação Física, Recife, PE, Brazil Universidade de Pernambuco Programa de Pós-graduação em Educação Física Recife PE Brazil; 3 Universidade Federal de Pernambuco, Recife, PE, Brazil Universidade Federal de Pernambuco Recife PE Brazil

**Keywords:** Adolescent, Adolescent Behavior, Violence, Risk Factors, Time Series Studies, Adolescente, Conducta del Adolescente, Violencia, Factores de Riesgo, Estudios de Series Temporales.

## Abstract

**Objective:**

To analyze the temporal trend and factors associated with indicators of physical violence among adolescents attending public high schools.

**Methods:**

A time series study with students aged 14 to 19 years, conducted at 5-year intervals. In 2006, 4,207 adolescents participated; in 2011, 6,264; in 2016, 6,002; and in 2022, 4,514. A modified version of the Global School-based Student Hfealth Survey was used. The dependent variables were physical violence and physical attacks. The independent variables were grouped into two hierarchical levels, in addition to risk behaviors (alcohol consumption, tobacco use, and illicit drug use). In the data analysis, multilevel binary logistic regression and segmented regression analysis were used.

**Results:**

A total of 20,987 adolescents participated. There was an increasing trend of 1.3% in physical violence and a reduction of −0.9% in physical attacks. Both showed reductions among males (−6.8 and −17.4, respectively) and increases among females (7.1 and 15.6, respectively). Exposure to alcohol, tobacco, and illicit drugs was identified as a factor associated with physical violence. Sex, race/skin color, alcohol consumption, and illicit drug use were found to be factors associated with physical attacks.

**Conclusion:**

Trends in risk factors associated with violence and physical attacks continue to include alcohol and illicit drugs, despite the decreasing trend in tobacco use for physical attacks. Male sex and race/skin color were the groups most exposed to physical attacks.

## Resumen

**Objetivo:**

Analizar la tendencia temporal y los factores asociados a los indicadores de violencia física en adolescentes de la educación secundaria de la red pública.

**Métodos:**

Estudio de series temporales con estudiantes de 14 a 19 años, realizado a intervalos de 5 años. En 2006 participaron 4.207 adolescentes; en 2011, 6.264; en 2016, 6.002; y en 2022, 4.514. Se utilizó una versión modificada del *Global School-based Student Health Survey*. Las variables dependientes fueron la violencia y los ataques físicos. Las variables independientes se agruparon en dos niveles jerárquicos, además de los comportamientos de riesgo (consumo de alcohol, tabaco y drogas ilícitas). En el análisis de datos se emplearon la regresión logística multinivel binaria y la regresión segmentada.

**Resultados:**

Participaron 20.987 adolescentes. Se observó una tendencia de aumento del 1,3 % en la violencia física y una disminución del −0,9 % en los ataques físicos. Ambos indicadores mostraron una reducción en el sexo masculino (−6,8 y −17,4, respectivamente) y un aumento en el sexo femenino (7,1 y 15,6, respectivamente). La exposición al alcohol, al tabaco y a las drogas ilícitas se identificó como factor asociado a la violencia física. Se verificó que el sexo, la raza o el color de piel, el consumo de alcohol y de drogas ilícitas son factores asociados a los ataques físicos.

**Conclusión:**

Las tendencias de los factores de riesgo asociados a la violencia y a los ataques físicos se mantienen en el ámbito del alcohol y las drogas ilícitas, a pesar de la tendencia decreciente del consumo de tabaco respecto a los ataques físicos. El sexo masculino y la raza/color de piel fueron los grupos más expuestos a ataques físicos.

**Ethical aspects**

This research respected ethical principles, having obtained the following approval data:

Research ethics committee: Hospital Agamenon Magalhães

Opinion number: -

Approval date: 1/7/2005

Certificate of submission for ethical appraisal: -

Research ethics committee: Universidade de Pernambuco

Opinion number: -

Approval date: 31/3/2011

Certificate of submission for ethical appraisal: 0158.0.097.000-10

Research ethics committee: Complexo Hospitalar HUOC/PROCAPE

Opinion number: 1.630.937

Approval date: 8/7/2016

Certificate of submission for ethical appraisal: 56341416.9.0000.5192

Research ethics committee: Fundação de Hematologia e Hemoterapia do Estado de Pernambuco

Opinion number: 4.449.705

Approval date: 24/9/2020

Certificate of submission for ethical appraisal: 38459320.3.0000.5195 Informed consent record: Obtained from all participants prior to data collection.

## Introduction 

Interpersonal violence during adolescence has devastating lifelong effects due to the magnitude of human rights violations, impacting physical, emotional, and social health^1^. It is subdivided into: i) intrafamilial (between partners and family members, but not exclusively within the domestic environment); ii) community (occurring in the social environment, between individuals without close or affective ties); and iii) collective (occurring at the macro-social, political, and economic level)^2^^,^^3^.

Exposure to violence has a significant impact on adolescent development, directly affecting how they interact socially and increasing the likelihood of adopting risk behaviors^4^. Physical violence frequently appears as an element associated with health risk behaviors. Studies indicate that alcohol consumption and violence constitute closely related risk behaviors, peaking during adolescence^5^^,^^6^.

Globally, interpersonal violence is the fifth leading cause of death among youth aged 10 to 24 years ^7^, and the second leading cause in low- and middle-income countries^8^^,^^9^. In 2022, for every 100 young people aged 15 to 29 who died in Brazil, 34 were homicide victims, and of the 46,409 homicides recorded, 49.2% were committed against young people^10^. Understanding the impact of interpersonal violence on adolescents and their participation as perpetrators is essential for identifying human rights violations and motivating actions/interventions by policymakers.

Expressions of violence are concentrated mainly in physical violence, characterized by the intentional use of force, such as slaps, pushes, kicks, pinches, and punches; and in physical attacks, defined as situations in which one or more people hit or strike another person, or use instruments such as knives or firearms^10^.

The World Health Organization (WHO) classifies a physical attack as occurring when an individual is forced to miss at least one full day of usual activities, such as studying, playing sports, or working, or when medical or nursing care is required. These injuries result in more than 5 million deaths annually and represent one of the most significant public health challenges^11^.

Adolescent exposure to violence and physical attacks has shown varying prevalence across countries. Between 2003 and 2017, there was an increase in the prevalence of violence among adolescents aged 13 to 15 years^12^^,^^13^^,^^14^. The National Survey of School Health (*Pesquisa Nacional de Saúde Escolar*, PeNSE) results are derived from a survey conducted with schoolchildren in early adolescence, and studies are scarce on late adolescents (14 years or older), who are transitioning to adulthood and entering the labor market. The objective of this study was to analyze the temporal trend and factors associated with indicators of exposure to physical violence among adolescents attending high school from 2006 to 2022. 

## Methods 

Study design

This was a time series study based on repeated cross-sectional surveys, using the same parameters, target population, sampling criteria, and periodicity (regular 5-year intervals: 2006, 2011, 2016, and 2022), except in 2021, when the continuation of the survey was hindered by the global emergency of acute respiratory infections associated with SARS-CoV-2.

Setting 

The state of Pernambuco is located in the Northeast region of Brazil and had an estimated population of 9,058,931 inhabitants in 2022^15^. It consists of 185 municipalities, distributed across 12 health regions and five geographic mesoregions: Metropolitan Region of Recife; Mata Pernambucana; Agreste Pernambucano; Sertão Pernambucano; and São Francisco Pernambucano^16^.

Participants 

Participants in this study were adolescents aged 14 to 19 years enrolled in public state high schools in Pernambuco, Brazil.

For participant selection, a two-stage cluster sampling method was used. In the first stage, schools were selected using stratified random sampling, stratified by size and geographic region. In the second stage, classes were selected based on shift distribution. In addition, a correction for a design effect was applied. In 2006, the design effect was 4%; in 2011, 2016, and 2022, it was 2%. To compensate for losses and refusals, the minimum sample size was increased by 20% in all surveys. The sample calculation considered a 95% confidence interval and an estimated prevalence of 50% for the variables^17^.

In the first survey, conducted in 2006, 76 public high schools were randomly selected. During data collection, students not aged 14 to 19 were excluded, leaving 4,207 students. In 2011, there were 85 schools with 6,264 students. In 2016, there were 6,002 students in 77 schools. Finally, in 2022, there were 4,514 students in 84 schools (Figure 1).

A translated, self-administered, and previously tested questionnaire, the Global School-based Student Health Survey, developed by WHO in collaboration with other international institutions, was used to assess adolescents’ exposure to health-risk behaviors.

Data collectors received prior training to guide respondents and clarify doubts. In addition, a pilot study was conducted to verify the instrument’s reproducibility and adequacy. The results indicated consistency between repeated measures (one-week interval), and adolescents generally found the questions easy to answer, despite the long participation time (approximately 30 minutes).

**Figure 1.**
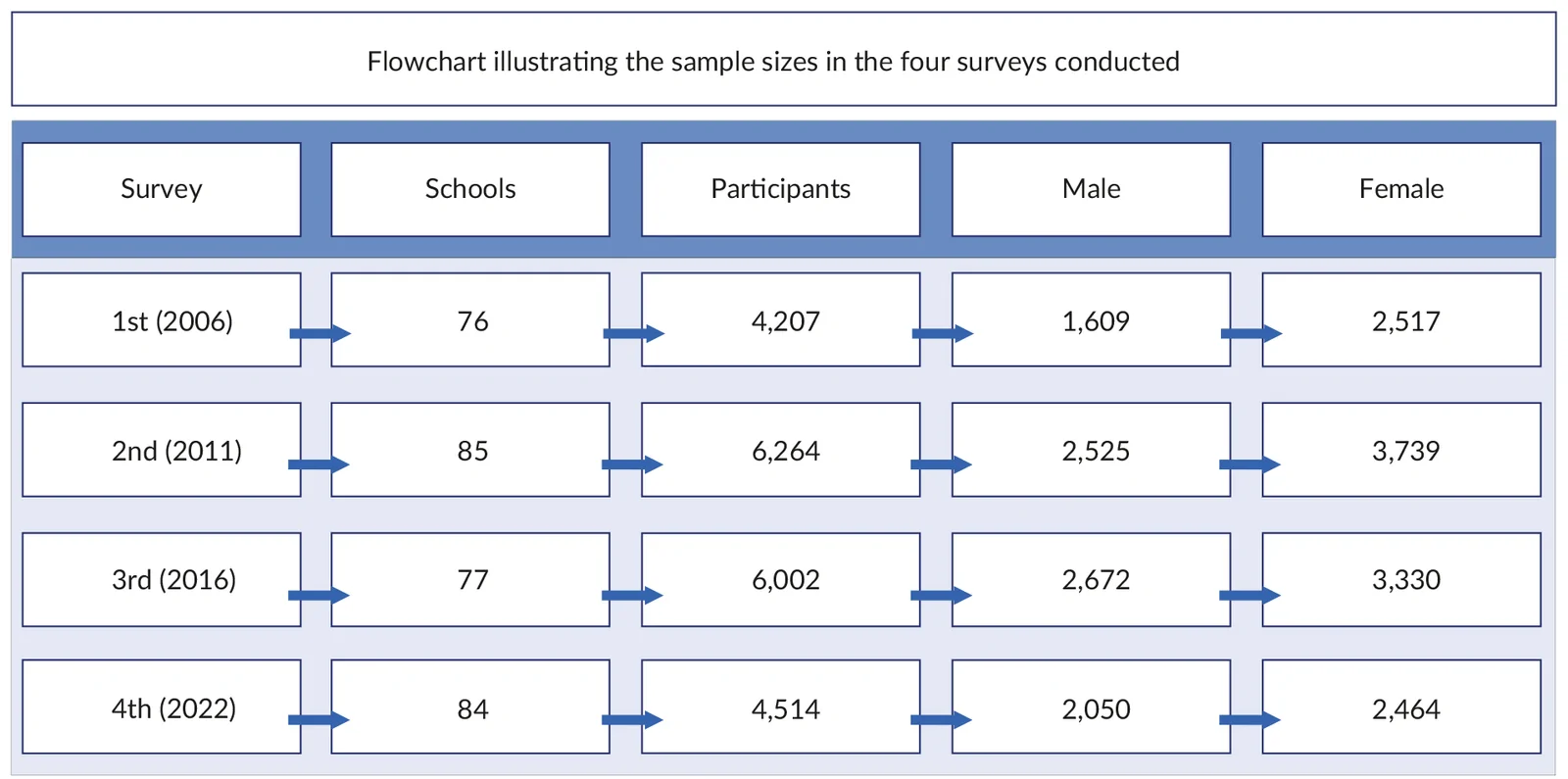
State surveys. Pernambuco, 2006, 2011, 2016, and 2022 (n=20,987)

Study variables

The dependent variables evaluated in this study were obtained through two questions:

Involvement in fights: “During the past 12 months, how many times have you been involved in a fight?”

Exposure to physical violence: “During the past 12 months, how many times have you experienced any type of physical violence?”

In both cases, respondents could choose one of the following response categories: i) none; ii) once; iii) 2 or 3 times; iv) 4 or 5 times; v) 6 or 7 times; vi) 8 or 9 times; vii) 10 or 11 times; and viii) 12 times or more. All students who reported any frequency greater than zero were considered cases of physical violence. 

The independent variables were grouped into two hierarchical levels (proximal and distal). This was done using an approach that guided the order in which variables were entered into the regression model. Therefore, in the regression analyses, variables at the distal level were included first, followed by the proximal level. The distal-level variables were: i) sex; ii) age; iii) race/skin color; and iv) maternal educational level. The proximal variables included risk behaviors, such as alcohol consumption in the past 30 days, tobacco use in the past 30 days, and illicit drug use in the past 30 days. 

Data sources

Data collection took place in the classroom, with an invitation extended to all students present. However, only those who presented a properly signed Informed Consent Form (ICF) by their parents and/or guardians and the Assent Form signed by the adolescents themselves were allowed to participate in the study. Participation was voluntary and occurred without teachers present to avoid potential intrusion effects.

Participants were informed about the study objectives, the data collection procedure, and that the information would be treated confidentially. In the first two surveys, the questionnaire was administered manually; in the last two, it was administered via electronic devices using the SPHINX software. At the end of each collection day, the data were synchronized and transferred to a single database, which, after cleaning and organization, could be used for analyses.

Statistical analysis

Data processing and analysis were performed using the Statistical Package for the Social Sciences (SPSS), version 25.0, and GraphPad Prism 6.0. The prevalence ratio (PR) and respective 95% confidence intervals (95%CI) for physical violence and physical attacks were calculated for all years assessed, according to sex.

The evolution was determined by comparing prevalence rates and their confidence intervals for 2006 and 2022, taking 2006 as the reference year. Temporal trends in the prevalence of violence and physical attacks were assessed and categorized by sex for each year of the study period using segmented regression analysis (version 5.2.0) ^18^.

From the slope of the regression line, the average annual percent change in rate evolution over the analyzed period was estimated. Trend measures over time were obtained, expressed as either positive or negative, along with the associated p-value.

Multilevel logistic regression models were performed, considering dependent and independent variables based on biological and sociodemographic plausibility. They were applied across all study years, with risk-behavior variables as the dependent variables in each year.

A final multivariate logistic regression analysis was conducted, including the collection years as an intervening variable to assess their influence on the model. Results were expressed as adjusted PR with their respective 95%CI with p-values<0.05 considered statistically significant.

## Results 

A total of 20,987 adolescents participated in the study across the four survey years: 4,207 in 2006, 6,264 in 2011, 6,002 in 2016, and 4,514 in 2022. The prevalence of physical violence remained above 8.0% in all years assessed, peaking in 2016 at 13.1%, while physical attacks reached 17.0%. There was an increasing trend of 1.3% in physical violence and a decrease of −0.9% in physical attacks (Figure 1). A reduction was observed among males and an increase among females. The average annual percent change was not statistically different for assaults and physical violence, even when categorized by sex (Supplementary Table 1).

Notably, in 2006, the prevalence of physical violence was 12.5% among males and 9.6% among females, whereas in 2022, the prevalence was 11.7% among males and 10.3% among females (Figure 2). Regarding physical attacks, in 2006, the prevalence was 28.0% among males and 16.6% among females, and in 2022, 23.2% among males and 19.2% among females (Figure 2). In all survey years, females predominated (Table 1). Adjusted and unadjusted logistic regression analyses showed that behavioral factors, such as alcohol consumption, tobacco use, and illicit drug use, remained associated as risk factors for physical violence (Table 2). 

Regarding physical attacks, male sex, race/skin color, alcohol consumption, and illicit drug use were considered risk factors in all survey years. Tobacco use, however, no longer appeared as a risk factor in the most recent survey year (Table 3). 

The logistic regression model was run, treating survey years as an intervening variable for assaults and violence, in addition to the independent variables [Table 2], to assess differences across years. For physical violence, 2006 was significantly different from 2011; for physical attacks, 2006 was significantly different from 2011, 2016, and 2022.

**Table 1:** Absolute and relative frequency n (%) of adolescents’ characteristics by sex, Pernambuco, 2006-2022 (n=20,987)

Variable	2006 (n=4,207)			2011 (n=6,264)			2016 (n=6,002)			2022 (n=4,514)		
	Male n (%)	Female n (%)	Total n (%)	Male n (%)	Female n (%)	Total n (%)	Male n (%)	Female n (%)	Total n (%)	Male n (%)	Female n (%)	Total n (%)
Age group (years)				
14-16	598 (35.4)	1,165 (46.4)	1,766 (42.0)	1,109 (43.9)	1,919 (51.3)	3,028 (48.3)	1,298 (48.6)	1,826 (55.4)	3,132 (52.2)	980 (47.8)	1,314 (53.3)	2,294 (50.8)
17-19	1,089 (64.6)	1,346 (53.6)	2,441 (58.0)	1,416 (56.1)	1,820 (48.7)	3,236 (51.7)	1,374 (51.4)	1,472 (44.6)	2,870 (47.8)	1,070 (52.2)	1,150 (46.7)	2,220 (49.2)
Race/skin color												
White	417 (24.8)	639 (25.5)	1,057 (25.2)	667 (26.5)	953 (25.6)	1,620 (25.9)	565 (21.2)	695 (21.1)	1,266 (21.2)	503 (24.5)	623 (25.3)	1,126 (24.9)
Black	884 (52.7)	1,194 (47.7)	2,085 (49.7)	386 (15.3)	404 (10.8)	790 (12.7)	436 (16.4)	369 (11.2)	812 (13.6)	376 (18.4)	335 (13.6)	711 (15.7)
Other^a^	378 (22.5)	672 (26.8)	1,051 (25.1)	1,463 (58.2)	2,369 (63.6)	3,832 (61.4)	1,658 (62.4)	2,225 (67.7)	3,902 (65.2)	1,171 (57.1)	1,506 (61.1)	2,677 (59.3)
Maternal educational level (years)												
<8	1,086 (69.4)	1,771 (74.5)	2,865 (72.5)	1,324 (60.5)	2,167 (67.6)	3,491 (64.7)	947 (42.7)	1,360 (48.7)	2,323 (46.1)	595 (35.5)	840 (41.1)	1,435 (38.5)
9-11	352 (22.5)	480 (20.2)	833 (21.1)	625 (28.6)	774 (24.1)	1,399 (26.0)	866 (38.9)	1,012 (36.3)	1,885 (37.4)	648 (38.6)	788 (38.5)	1,436 (38.6)
≥12	127 (8.1)	126 (5.3)	253 (6.4)	239 (10.9)	265 (8.3)	504 (9.3)	410 (18.4)	419 (15.0)	835 (16.5)	435 (25.9)	418 (20.4)	853 (22.9)

a Brown (Brazilian mixed race), Asian, and Indigenous.

**Figure 2.**
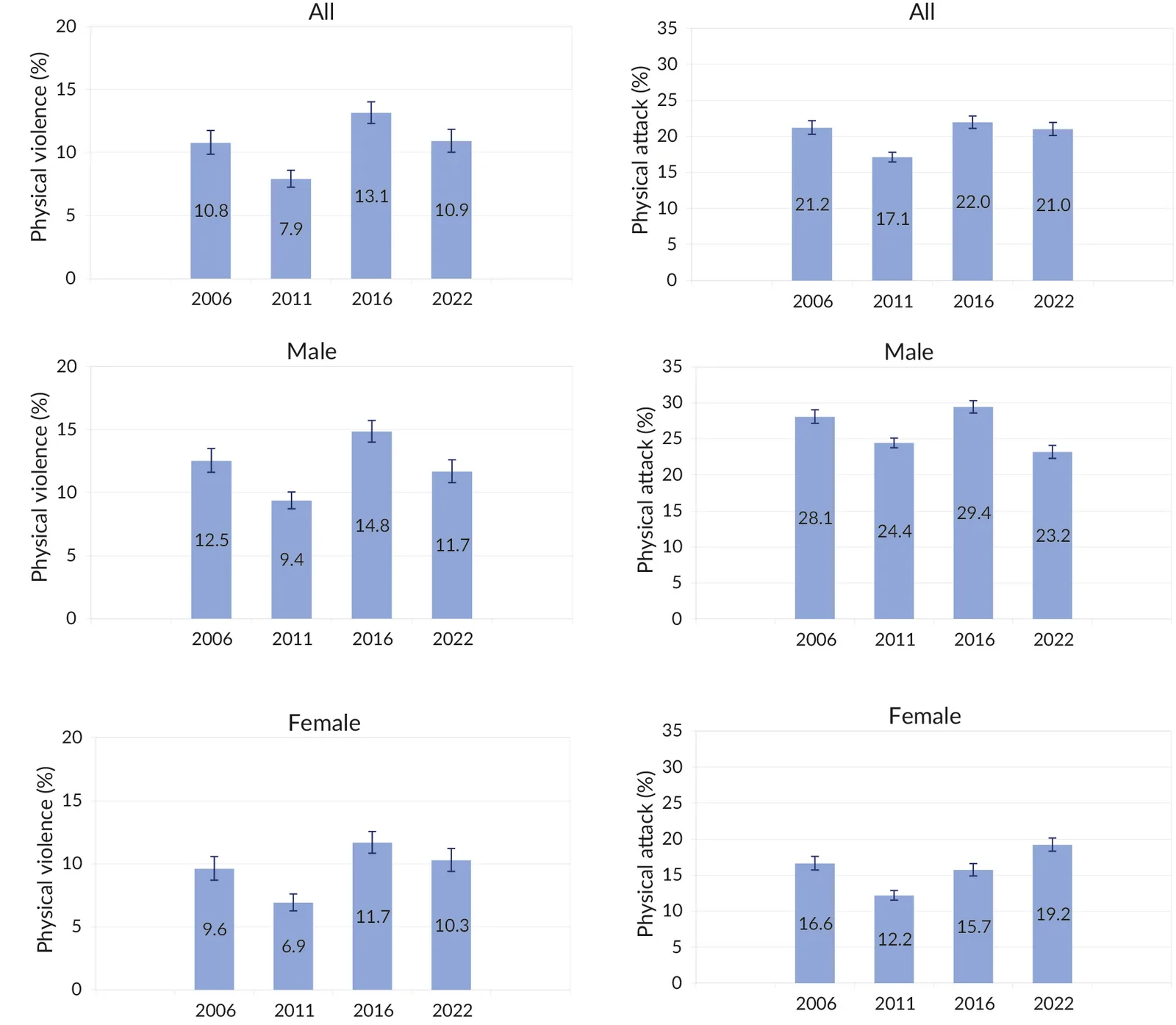
Prevalence and 95% confidence interval (95%CI) and temporal trends of physical violence and physical attacks among school adolescents. Pernambuco, 2006, 2011, 2016, and 2022 (n=20,987)

**Table 2:** Odds ratio (OR) and 95% confidence intervals (95%CI) adjusted for physical violence according to study variables. Pernambuco, 2006-2022 (n=20,987)

Variables	2006			2011		2016		2022	
	OR (95%CI)	p-value^a^	OR (95%CI)	p-value^a^	OR (95%CI)	p-value^a^	OR (95%CI)	p-value^a^
Individual and family factors				
Sex								
Female	1		1		1		1	
Male	1.10 (0.89; 1.37)	0.350	1.29 (1.05; 1.58)	0.014	1.21 (1.02; 1.44)	0.024	1.08 (0.87; 1.34)	0.453
Age (years)								
14	1		1				1	
15	0.91 (0.51; 1.62)	0.755	1.24 (0.70; 2.18)	0.451	0.95 (0.57; 1.59)	0.862	1.37 (0.57; 3.30)	0.474
16	1.07 (0.62; 1.85)	0.801	1.03 (0.59; 1.79)	0.906	0.90 (0.55; 1.50)	0.709	1.16 (0.48; 2.78)	0.727
17	0.84 (0.48; 1.45)	0.533	0.87 (0.50; 1.53)	0.649	1.00 (0.60; 1.65)	0.993	0.90 (0.37; 2.16)	0.817
18	1.05 (0.60; 1.83)	0.858	0.92 (0.51; 1.63)	0.776	0.80 (0.47; 1.36)	0.418	0.90 (0.37; 2.18)	0.815
19	0.77 (0.42; 1.41)	0.407	0.97 (0.52; 1.81)	0.934	0.92 (0.50; 1.71)	0.814	0.72 (0.26; 2.01)	0.539
Race/skin color								
White	1		1		1		1	
Black	1.12 (0.88; 1.43)	0.344	1.17 (0.91; 1.50)	0.210	1.09 (0.88; 1.35)	0.409	0.85 (0.67; 1.08)	0.208
Other^b^	1.10 (0.66; 1.84)	0.695	1.68 (1.20; 2.36)	0.002	1.19 (0.88; 1.61)	0.237	1.23 (0.78; 1.92)	0.367
Maternal educational level								
No formal education	1		1		1		1	
Complete primary education	0.91 (0.68; 1.23)	0.577	0.98 (0.67; 1.44)	0.951	1.64 (1.01; 2.67)	0.044	0.77 (0.42; 1.39)	0.393
Complete secondary education	1.09 (0.76; 1.57)	0.623	1.06 (0.69; 1.63)	0.763	1.85 (1.14; 3.00)	0.012	0.99 (0.54; 1.81)	0.990
Complete secondary education or higher	0.93 (0.66; 1.31)	0.703	0.87 (0.58; 1.30)	0.504	1.74 (1.09; 2.78)	0.020	0.92 (0.52; 1.64)	0.793
Behavioral factors								
Alcohol consumption								
No	1		1		1		1	
Yes	1.60 (1.25; 2.00)	<0.001	1.34 (1.07; 1.67)	0.010	1.40 (1.16; 1.67)	<0.001	1.61 (1.27; 2.03)	<0.001
Tobacco use								
No	1		1		1		1	
Yes	1.47 (1.04; 2.07)	0.025	2.59 (1.84; 3.66)	<0.001	1.62 (1.22; 2.16)	0.001	1.57 (1.10; 2.24)	0.012
Illicit drug use								
No	1		1		1		1	
Yes	2.18 (1.57; 3.03)	<0.001	2.08 (1.51; 2.86)	<0.001	1.88 (1.47; 2.40)	<0.001	2.56 (1.86; 3.52)	<0.001

a Logistic regression adjusted for the variables included in the table; ^b^Brown (Brazilian mixed race), Asian, and Indigenous.

**Table 3:** Odds ratio (OR) and adjusted 95% confidence intervals (95%CI) of physical attacks for the study variables. Pernambuco, 2006-2022 (n=20,987)

Variables	2006			2011		2016		2022	
	OR (95%CI)	p-value^a^	OR (95%CI)	p-value^a^	OR (95%CI)	p-value^a^	OR (95%CI)	p-value^a^
Individual and family factors								
Sex								
Female	1^b^		1		1		1	
Male	1.44 (1.17; 1.77)	<0.001	2.33 (2.01; 2.71)	<0.001	2.17 (1.88; 2.51)	<0.001	1.24 (1.05; 1.47)	0.008
Age (years)								
14	1		1		1		1	
15	2.76 (1.05; 7.29)	0.040	0.98 (0.65; 1.48)	0.956	1.02 (0.65; 1.59)	0.919	1.33 (0.69; 2.58)	0.391
16	3.04 (1.18; 7.85)	0.021	0.95 (0.64; 1.42)	0.832	0.87 (0.56; 1.35)	0.557	1.13 (0.59; 2.19)	0.697
17	3.96 (1.55; 10.1)	0.004	0.86 (0.58; 1.28)	0.462	0.96 (0.62; 1.48)	0.855	0.90 (0.46; 1.74)	0.766
18	4.73 (1.84; 12.1)	0.001	0.80 (0.53; 1.22)	0.311	0.80 (0.50; 1.26)	0.342	0.87 (0.44; 1.70)	0.688
19	5.07 (1.94; 13.2)	0.001	0.79 (0.50; 1.25)	0.327	0.51 (0.29; 0.89)	0.018	1.15 (0.54; 2.43)	0.703
Race/skin color								
White	1		1		1		1	
Black	1.11 (0.87; 1.41)	0.397	0.96 (0.81; 1.15)	0.704	0.95 (0.79; 1.13)	0.576	1.28 (1.05; 1.50)	0.012
Other^b^	1.30 (0.80; 2.12)	0.280	1.22 (0.94; 1.57)	0.126	1.23 (0.96; 1.58)	0.101	1.95 (1.36; 2.77)	<0.001
Maternal educational level								
No formal education	1		1		1		1	
Complete primary education	0.92 (0.68; 1.23)	0.592	0.84 (0.64; 1.11)	0.240	1.36 (0.93; 1.99)	0.109	0.97 (0.60; 1.56)	0.908
Complete secondary education	0.79 (0.54; 1.16)	0.239	0.84 (0.61; 1.15)	0.296	1.64 (1.13; 2.39)	0.009	1.09 (0.67; 1.77)	0.710
Complete secondary education or higher	1.19 (0.80; 1.54)	0.496	0.92 (0.69; 1.23)	0.597	1.35 (0.94; 1.95)	0.099	1.14 (0.72; 1.81)	0.560
Behavioral factors								
Alcohol consumption								
No	1		1		1		1	
Yes	4.26 (3.44; 5.28)	<0.001	1.81 (1.54; 2.13)	<0.001	2.02 (1.74; 2.36)	<0.001	1.91 (1.59; 2.29)	<0.001
Tobacco use								
No	1		1		1		1	
Yes	1.56 (1.15; 2.12)	0.004	2.13 (1.60; 2.83)	<0.001	2.31 (1.80; 2.96)	<0.001	1.27 (0.93; 1.72)	0.121
Illicit drug use								
No	1		1		1		1	
Yes	3.72 (2.77; 5.00)	<0.001	2.35 (1.84; 3.01)	<0.001	1.95 (1.57; 2.42)	<0.001	2.14 (1.63; 2.81)	<0.001

a Logistic regression; ^b^ Brown (Brazilian mixed race), Asian, and Indigenous.

## Discussion 

Adolescents attending Brazilian public schools exhibit a high prevalence of physical violence and physical attacks, with the highest rates observed in 2016. A decrease was observed among males and an increase among females, so that, in the most recent year assessed, the prevalence of physical violence was similar and approached that of physical attacks. Exposure to alcohol and drugs remained a risk factor across all years evaluated. However, tobacco use was no longer associated with physical attacks in the last survey year. Male sex continued to be more exposed to assaults, and race/skin color remained a risk factor.

As a limitation, this study did not capture the entire adolescent population, particularly those not attending school. The results should not be generalized to the whole adolescent population. Response biases, recall biases, and social desirability may reduce self-reported experiences of violence and health risk behaviors. 

The increase in the prevalence of adolescents who are victims of violence highlights the need for continuous support and public policies^19^. Rights violations, whether direct or indirect, impact physical and mental health, as well as academic performance, social relationships, and family interactions^20^. Associations were observed with behaviors and profiles such as being male, using legal and illicit drugs, skipping classes, residing in violent areas, facing family conflicts, experiencing insufficient parental supervision, and having depression ^21^^,^^22^.

The Health Behavior in School-aged Children (HBSC) study found that boys are more likely than girls to be involved in fights. Furthermore, this gender-related variation was noted in nearly all countries analyzed ^20^. Violent events were more prevalent in public institutions and among male students^12^.

This study showed that both physical violence and physical attacks decreased among males and increased among females. These patterns are considered to be related to social constructs, grounded in culturally ingrained notions of masculinity, strength, power dynamics, and the normalization and acceptance of violence^19^^,^^20^^,^^23^. Therefore, there is a trend toward convergence in aggressive behaviors between genders, likely influenced by sociocultural, environmental, and technological factors^24^.

The vulnerability observed among females reflects historical and cultural processes marked by sexism and patriarchy. Between 2015 and 2016, women launched the hashtag “#vamosjuntas?” to build supportive networks through social media, aiming to prevent violence in public spaces and foster collaboration among women, who have higher Internet access than men^24^.

Virtual platforms are essential in the current context, allowing the expansion, dissemination, and coordination of ideas within social movements. Additionally, they aim to restore power relations, restructure interests, and promote social transformation ^25^. Therefore, it is necessary to strengthen the implementation of public policies and violence-prevention programs to reduce social disparities and to reinforce legislation.

Exposure to alcohol, tobacco, and illicit substances persisted as a risk factor for physical violence. Regarding physical attacks, male sex, race/skin color, alcohol consumption, and illicit drug use were risk factors. In contrast, tobacco use no longer appeared as a risk factor in the last survey year. In this context, cigarette consumption among adolescents decreased. However, alcohol and other drug consumption increased over the years and was associated with violent behaviors^26^^,^^27^.

This study analyzed the predictors of alcohol and illicit drug use and violence among adolescents. Nevertheless, the complexity in determining the order of these behaviors has made it difficult to define causal relationships among them ^28^. Substance use was associated with a 2.4-fold higher likelihood of involvement in violence among youth ^29^. Alcohol consumption is associated with impaired judgment, reduced self-control, and an increased likelihood of risk-taking behaviors and violence^30^.

In this context, it is essential to plan and evaluate public policies to prevent violence, promote health, and foster a culture of peace. Moreover, professionals need training to identify situations of violence and to integrate with social assistance and support networks.

## Data Availability

The database and analysis code used in this research are available upon request, with access granted upon justification through the corresponding author.
