# Supplementary material for: Indicators of exposure to physical violence among adolescents, Pernambuco, 2006-2022: time series
Source: Epidemiol Serv Saude. 2026 May 13;35:e20250215. doi: 10.1590/S2237-96222026v35e20250215.en (PMC13686416; doi:10.1590/S2237-96222026v35e20250215.en)
Supplement: Supplementary file 1 [file 2237-9622-ress-35-e20250215-tabela-suplementar-EN.pdf]

Supplementary Table 1. Temporal trend from 2006 to 2022 and average annual percent change (AAPC) with the corresponding 95% confidence interval of the prevalence of physical violence and physical assault among school-aged adolescents. Pernambuco, 2006, 2011, 2016, and 2022 (n=20,987)

**Variable**	All			Male			Female		
	%	95%CI	p-value	%	95%CI	p-value	%	95%CI	p-value
**Temporal trend from 2006 to 2022**									
Physical violence	1.3	-	-	-6.8	-	-	7.1	-	-
Physical attack	-0.9	-	-	-17.4	-	-	15.6	-	-
**AAPC**									
Physical violence	1.0	-4.13; 6.27	0.666	0.4	-5.34; 6.42	0.845	1.4	-2.92; 5.81	0.563
Physical attack	0.4	-1.44; 2.25	0.694	-0.8	-3.43; 1.83	0.514	1.4	-2.66; 5.57	0.506
