# Supplementary material for: Indicators of exposure to physical violence among adolescents, Pernambuco, 2006-2022: time series
Source: Epidemiol Serv Saude. 2026 May 13;35:e20250215. doi: 10.1590/S2237-96222026v35e20250215.en (PMC13686416; doi:10.1590/S2237-96222026v35e20250215.en)
Supplement: Supplementary file 2 [file 2237-9622-ress-35-e20250215-tabela-suplementar-PT.pdf]

Tabela suplementar 1. Evolução temporal 2006 a 2022 e variação percentual média anual (VPMA) e respectivo Intervalo de Confiança 95% da prevalência de violência física e ataque físico em adolescentes escolares. Pernambuco, 2006, 2011, 2016 e 2022 (n=20.987)

**Variável**	Todos			Masculino			Feminino		
	%	IC95%	p-valor	%	IC95%	p-valor	%	IC95%	p-valor
**Evolução temporal 2006 a 2022**									
Violência física	1,3	-	-	-6,8	-	-	7,1	-	-
Ataque físico	-0,9	-	-	-17,4	-	-	15,6	-	-
**VPMA**									
Violência física	1,0	-4,13; 6,27	0,666	0,4	-5,34; 6,42	0,845	1,4	-2,92; 5,81	0,563
Ataque físico	0,4	-1,44; 2,25	0,694	-0,8	-3,43; 1,83	0,514	1,4	-2,66; 5,57	0,506
