# Supplementary material for: Indicators of exposure to physical violence among adolescents, Pernambuco, 2006-2022: time series
Source: Epidemiol Serv Saude. 2026 May 13;35:e20250215. doi: 10.1590/S2237-96222026v35e20250215.en (PMC13686416; doi:10.1590/S2237-96222026v35e20250215.en)
Supplement: Translation

# Translation: Indicadores de exposição à violência física em adolescentes, Pernambuco, 2006-2022: série temporal

**DOI:** 10.1590/S2237-96222026v35e20250215.pt

**Authors:** Cláudia Fabiane Gomes Gonçalves, Mauro Virgílio Gomes Barros, Lygia Maria Pereira da Silva, Marco Aurélio de Valois Correia Junior, Emilia Chagas Costa, Caroline Ramos de Moura Silva

**Affiliations:** 1 Universidade de Pernambuco, Programa de Pós-graduação em Hebiatria, Recife, PE, Brasil Universidade de Pernambuco Programa de Pós-graduação em Hebiatria Recife PE Brasil; 2 Universidade de Pernambuco, Programa de Pós-graduação em Educação Física, Recife, PE, Brasil Universidade de Pernambuco Programa de Pós-graduação em Educação Física Recife PE Brasil; 3 Universidade Federal de Pernambuco, Recife, PE, Brasil Universidade Federal de Pernambuco Recife PE Brasil

**Keywords:** Adolescente, Comportamento do Adolescente, Violência, Fatores de Risco, Estudos de Séries Temporais.

## Abstract

**Objetivo:**

Analisar a tendência temporal e os fatores associados a indicadores de violência física em adolescentes, estudantes do ensino médio da rede pública.

**Métodos:**

Estudo de séries temporais com estudantes de 14 a 19 anos, conduzido em intervalos de 5 anos. Em 2006, participaram 4.207 adolescentes; em 2011, 6.264; em 2016, 6.002; e, em 2022, 4.514. Utilizou-se versão modificada do *Global School-based Student Health Survey*. As variáveis dependentes foram violência física e ataques físicos. As variáveis independentes foram agrupadas em dois níveis hierárquicos, além dos comportamentos de risco (consumo de álcool, de tabaco e de drogas ilícitas). Na análise de dados, utilizaram-se a regressão logística binária multinível e a análise de regressão segmentada.

**Resultados:**

Ao todo, participaram 20.987 adolescentes. Houve tendência de aumento de 1,3% na violência física e uma redução de -0,9% nos ataques físicos. Ambos tiveram redução no sexo masculino (-6,8 e -17,4, respectivamente) e aumento no feminino (7,1 e 15,6, respectivamente). A exposição ao álcool, ao tabaco e às drogas ilícitas foram identificados como fatores associados à violência física. Verificou-se que sexo, raça/cor da pele, consumo de álcool e de drogas ilícitas são fatores associados aos ataques físicos.

**Conclusão:**

As tendências dos fatores de risco associados à violência e aos ataques físicos permanecem o álcool e as drogas ilícitas, apesar da tendência decrescente do tabagismo para ataque físico. O sexo masculino e a raça/cor da pele foram os grupos mais expostos a ataques físicos.

**Aspectos éticos**

Esta pesquisa respeitou os princípios éticos, obtendo os seguintes dados de aprovação:

Comitê de ética em pesquisa: Hospital Agamenon Magalhães

Número do parecer: -

Data de aprovação: 1/7/2005

Certificado de apresentação de apreciação ética: -

Comitê de ética em pesquisa: Universidade de Pernambuco

Número do parecer: -

Data de aprovação: 31/3/2011

Certificado de apresentação de apreciação ética: 0158.0.097.000-10

Comitê de ética em pesquisa: Complexo Hospitalar HUOC/PROCAPE

Número do parecer: 1.630.937

Data de aprovação: 8/7/2016

Certificado de apresentação de apreciação ética: 56341416.9.0000.5192

Comitê de ética em pesquisa: Fundação de hematologia e hemoterapia do estado de Pernambuco

Número do parecer: 4.449.705

Data de aprovação: 24/9/2020

Certificado de apresentação de apreciação ética: 38459320.3.0000.5195

Registro do consentimento livre e esclarecido: Obtido de todos os participantes antes da coleta.

## Introdução 

A violência interpessoal na adolescência possui efeitos devastadores ao longo da vida, devido à magnitude na violação aos direitos humanos, com impactos na saúde física, emocional e social^1^. Subdivide-se em: i) intrafamiliar (entre parceiros e membros da família, mas não exclusivamente dentro do ambiente doméstico); ii) comunitária (ocorrida no ambiente social, entre indivíduos que não possuem proximidade ou laço afetivo); e iii) coletiva (ocorrida no âmbito macrossocial, político e econômico)^2^^,^^3^.

A exposição à violência tem impacto significativo sobre o desenvolvimento dos adolescentes, afetando diretamente a maneira como interagem socialmente e aumentando a chance de adotarem comportamentos de risco^4^. A violência física se apresenta como um elemento frequentemente associado a comportamentos de risco à saúde. Estudos indicam que o consumo de bebidas alcoólicas e a violência constituem comportamentos de risco que estão intimamente relacionados, apresentando um pico de incidência na adolescência^5^^,^^6^.

No mundo, a violência interpessoal é a quinta principal causa de óbito entre jovens de 10 a 24 anos^7^, e a segunda principal causa em países de média e baixa renda^8^^,^^9^. No ano de 2022, a cada 100 jovens de 15 a 29 anos que morreram no Brasil, 34 foram vítimas de homicídio, e, dos 46.409 homicídios contabilizados, 49,2% foram cometidos contra jovens^10^. Compreender o impacto da violência interpessoal em adolescentes e sua participação como autores de violência é fundamental para identificar violações de direitos humanos e motivar ações/intervenções por parte dos gestores.

As expressões da violência se concentram, sobretudo, na violência física, caracterizada pelo uso intencional da força, como tapas, empurrões, chutes, beliscões e socos; e nos ataques físicos, que podem ser definidos quando uma ou mais pessoas batem ou golpeiam outra, ou quando utilizam mecanismos, como arma branca ou arma de fogo^10^.

A Organização Mundial de Saúde (OMS), classifica ataque físico quando o indivíduo é forçado a perder pelo menos um dia inteiro de atividades habituais, como estudar, praticar esportes ou trabalhar, ou necessita de assistência médica ou de enfermagem. Esses ferimentos resultam em mais de 5 milhões de óbitos anualmente e configuram um dos mais relevantes entraves no âmbito da saúde pública.^11^.

A exposição de adolescentes à violência e ataque físico tem apresentado prevalências distintas entre países. Entre 2003 e 2017, houve crescimento na prevalência da violência entre adolescentes de 13 a 15 anos^12^^,^^14^. Os resultados da Pesquisa Nacional da Saúde Escolar (PeNSE) são derivados de levantamentos realizados com escolares no início da adolescência, havendo escassez de estudos com adolescentes tardios (14 anos ou mais), os quais se encontram na transição para vida adulta e ingresso no mercado de trabalho. O objetivo deste estudo foi analisar a tendência temporal e os fatores associados a indicadores de exposição à violência física em adolescentes, estudantes do ensino médio, no período de 2006 a 2022. 

## Métodos 

Delineamento do estudo

Estudo de série temporal baseado em inquéritos transversais repetidos, com os mesmos parâmetros, população-alvo, critérios amostrais e periodicidade (intervalos regulares de cinco anos: 2006, 2011, 2016 e 2022), exceto no ano de 2021, em que a continuidade do inquérito foi inviabilizada pela emergência global de infecções respiratórias agudas associadas ao SARS-CoV-2.

Contexto 

O estado de Pernambuco está localizado na região Nordeste do Brasil e possuía uma população estimada de 9.058.931 habitantes no ano de 2022^15^. É constituído por 185 municípios, distribuídos em 12 regiões de saúde e cinco mesorregiões geográficas: Metropolitana do Recife; Mata Pernambucana; Agreste Pernambucano; Sertão Pernambucano e São Francisco Pernambucano^16^.

Participantes 

Os participantes deste estudo foram adolescentes, com idade entre 14 e 19 anos, matriculados em escolas pública estaduais de ensino médio de Pernambuco, Brasil.

Para a seleção dos participantes, utilizou-se uma amostragem por conglomerados em dois estágios. No primeiro estágio, a escola foi escolhida por amostragem aleatória estratificada, levando em conta a distribuição por porte e região geográfica. No segundo estágio, as turmas foram selecionadas considerando a distribuição por turno. Além disso, foi aplicada correção de efeito de delineamento. Em 2006, o efeito de delineamento adotado foi 4%, enquanto em 2011, 2016 e 2022 foi 2%. Para compensar perdas e recusas, a amostra mínima foi inflada em 20% em todos os inquéritos. O cálculo amostral considerou intervalo de confiança de 95% e prevalência estimada das variáveis de 50%^17^.

No primeiro levantamento, realizado em 2006, foram sorteadas 76 escolas públicas de ensino médio. Ao longo da coleta de dados, foram excluídos os estudantes que não tinham entre 14 e 19 anos, restando 4.207 estudantes. Em 2011, foram 85 escolas, com 6.264 estudantes. Em 2016, foram 6.002 estudantes, em 77 escolas. Por fim, em 2022, foram 4.514 estudantes, em 84 escolas (Figura 1).

Utilizou-se um questionário traduzido, autoadministrado e previamente testado, proposto pela OMS, em colaboração com outras instituições internacionais, o *Global School-based Student Health Survey*. que tem como objetivo mensurar a exposição de adolescentes a comportamentos relacionados ao risco à saúde.

Os aplicadores receberam treinamento prévio ao início da coleta de dados, que guiaram os respondentes a fim de esclarecer dúvidas. Além disso, realizou-se um estudo piloto para verificar a reprodutibilidade das medidas e a adequação do instrumento. Os resultados indicaram consistência entre medidas repetidas (intervalo de uma semana) e os adolescentes acharam as perguntas, em geral, fáceis de responder, apesar do tempo de participação ser longo (cerca de 30 minutos).

**Figura 1:** Inquéritos estaduais. Pernambuco, 2006, 2011, 2016 e 2022 (n=20.987)

Variáveis do estudo

As variáveis dependentes avaliadas neste estudo foram retiradas por meio de duas questões:

Envolvimento em brigas: “Durante os últimos 12 meses, quantas vezes você esteve envolvido em uma luta?”

Exposição à violência física: “Durante os últimos 12 meses, quantas vezes você sofreu algum tipo de violência física?”

Nos dois casos, os respondentes poderiam informar uma das seguintes categorias de resposta: i) nenhuma vez; ii) 1 vez; iii) 2 ou 3 vezes; iv) 4 ou 5 vezes; v) 6 ou 7 vezes; vi) 8 ou 9 vezes; vii) 10 ou 11 vezes; e viii) 12 vezes ou mais. Todos os estudantes que referiram qualquer frequência diferente de zero foram considerados casos de violência física. 

As variáveis independentes foram agrupadas em dois níveis hierárquicos (proximal e distal). Isso foi feito por meio de abordagem que orientou a ordem de entrada das variáveis no modelo de regressão. Desse modo, nas análises de regressão foram incluídas primeiro as variáveis no nível distal, e em seguida no proximal. As variáveis do nível distal foram: i) sexo; ii) idade; iii) raça/cor da pele; e iv) escolaridade materna. Já as variáveis proximais abrangeram os comportamentais de risco, como consumo de álcool nos últimos 30 dias, consumo de tabaco nos últimos 30 dias, e uso de drogas ilícitas nos últimos 30 dias. 

Fonte de dados

A etapa de coleta ocorreu em sala de aula, com convite estendido a todos os estudantes presentes. Porém, apenas os que apresentaram o Termo de Consentimento Livre e Esclarecido (TCLE) devidamente assinado pelos pais e/ou responsáveis e o Termo de Assentimento Livre e Esclarecido, assinado pelos adolescentes, foram autorizados a integrar o estudo. A participação era de forma voluntária, sem a presença dos professores, a fim de evitar possível efeito de intrusão.

Os participantes foram informados sobre os objetivos da pesquisa e o dispositivo de coleta de dados e que as informações seriam tratadas de forma confidencial. Nos primeiros dois inquéritos, o questionário foi aplicado manualmente; nos últimos dois, em dispositivos eletrônicos com o software SPHINX. Ao final do dia de coleta, os dados foram sincronizados e transferidos para uma base única, que, após ser submetido a limpeza e organização, poderia ser utilizado para as análises.

Análise estatística

O processamento e a análise dos dados foram realizados utilizando o Statistical Package for the Social Sciences (SPSS), versão 25.0, e o GraphPad Prism 6.0. A razão de prevalência (RP) e os respectivos intervalos de confiança de 95% (IC95%) para violência e ataque físico foram calculados em todos os anos avaliados, de acordo com o sexo.

A evolução foi determinada pela comparação das taxas de prevalência e seus intervalos de confiança para 2006 e 2022, tomando 2006 como ano de referência. A avaliação das tendências temporais para a prevalência da violência e ataque físico foi calculada e categorizada por sexo para cada ano do período estudado e foi realizada usando a análise de regressão segmentada, versão 5.2.0^18^.

A partir da inclinação da reta, estimou-se a variação percentual média anual na evolução das taxas durante o período analisado. Obtiveram-se medidas de tendência ao longo do tempo, expressas em negativa ou positiva, e o p-valor associado.

Modelos de regressão logística multinível foram realizados, considerando variáveis dependentes e independentes baseadas em plausibilidade biológica e sociodemográfica, aplicadas em todos os anos, incluindo variáveis de comportamento de risco como dependentes em todos os anos estudados.

Foi realizada última análise de regressão logística multivariada, incluindo os anos de coleta como variável interveniente para avaliar a influência dos anos das coletas no modelo. Os resultados foram expressos como RP ajustadas acompanhadas de seus respectivos IC95%, considerando-se significativos os p-valores inferiores a 0,05.

## Resultados 

Participaram da pesquisa 20.987 adolescentes, somando-se os quatro anos dos inquéritos, com 4.207 em 2006, 6.264 em 2011, 6.002 em 2016 e 4.514 em 2022. A prevalência da violência física permaneceu acima dos 8,0% em todos os anos avaliados, com ápice em 2016, com 13,1%, e de ataque físico em 17,0%. Houve tendência de aumento de 1,3% da violência física e uma diminuição de -0,9% para ataque físico (Figura 1). Observou-se redução no sexo masculino e aumento no feminino. A variação percentual média anual não foi estatisticamente diferente para agressões e violência física, mesmo quando categorizada por sexo (Tabela suplementar Tabela 1).

Destaca-se que, em 2006, para violência física, o sexo masculino teve prevalência de 12,5% e o sexo feminino de 9,6%, enquanto em 2022 a prevalência no masculino foi 11,7% e no feminino 10,3% (Figura 2). Com relação ao ataque físico, em 2006, o sexo masculino obteve prevalência de 28,0% e o sexo feminino de 16,6%, e em 2022, o masculino teve prevalência de 23,2% e o feminino de 19,2% (Figura 2). Em todas as coletas, o sexo feminino prevaleceu (Tabela 1). As análises de regressão logística bruta e ajustada evidenciaram que os fatores comportamentais, como consumo de álcool, de tabaco e de drogas ilícitas, permaneceram associados como fatores de risco para violência física (Tabela 2). 

Quanto ao ataque físico, os fatores sexo masculino, raça/cor da pele, consumo de álcool e de drogas ilícitas foram considerados de risco em todos os anos da coleta. Já o consumo de tabaco deixou de apresentar-se como risco no último ano da coleta (Tabela 3). 

O modelo de regressão logística foi rodado considerando os anos como uma variável interveniente para agressões e violência, além das variáveis independentes (Tabela 2), com o objetivo de avaliar diferenças entre os anos. Quanto à violência, o ano de 2006 foi significativamente diferente do ano 2011, enquanto o ano de 2006 foi significativamente diferente de 2011, 2016 e 2022 quanto às agressões.

**Tabela 1:** Frequência absoluta e relativa n (%) das características dos adolescentes de acordo com o sexo, Pernambuco, 2006-2022 (n=20.987)

Variável	2006 (n=4.207)			2011 (n=6.264)			2016 (n=6.002)			2022 (n=4.514)		
	Masculino n (%)	Feminino n (%)	Total n (%)	Masculino n (%)	Feminino n (%)	Total n (%)	Masculino n (%)	Feminino n (%)	Total n (%)	Masculino n (%)	Feminino n (%)	Total n (%)
Faixa etária (anos)				
14-16	598 (35,4)	1.165 (46,4)	1.766 (42,0)	1.109 (43,9)	1.919 (51,3)	3.028 (48,3)	1.298 (48,6)	1.826 (55, 4)	3.132 (52,2)	980 (47,8)	1.314 (53,3)	2.294 (50,8)
17-19	1.089 (64,6)	1.346 (53,6)	2.441 (58,0)	1.416 (56,1)	1.820 (48,7)	3.236 (51,7)	1.374 (51,4)	1.472 (44,6)	2.870 (47,8)	1.070 (52,2)	1.150 (46,7)	2.220 (49,2)
Raça/cor da pele												
Branca	417 (24,8)	639 (25,5)	1.057 (25,2)	667 (26,5)	953 (25,6)	1.620 (25,9)	565 (21,2)	695 (21,1)	1.266 (21,2)	503 (24,5)	623 (25,3)	1.126 (24,9)
Preta	884 (52,7)	1.194 (47,7)	2.085 (49,7)	386 (15,3)	404 (10,8)	790 (12,7)	436 (16,4)	369 (11,2)	812 (13,6)	376 (18,4)	335 (13,6)	711 (15,7)
Outrasa	378 (22,5)	672 (26,8)	1.051 (25,1)	1.463 (58,2)	2.369 (63,6)	3.832 (61,4)	1.658 (62,4)	2.225 (67,7)	3.902 (65,2)	1.171 (57,1)	1.506 (61,1)	2.677 (59,3)
Escolaridade materna (anos)												
<8	1.086 (69,4)	1.771 (74,5)	2.865 (72,5)	1.324 (60,5)	2.167 (67,6)	3.491 (64,7)	947 (42,7)	1.360 (48,7)	2.323 (46,1)	595 (35,5)	840 (41,1)	1.435 (38,5)
9-11	352 (22,5)	480 (20,2)	833 (21,1)	625 (28,6)	774 (24,1)	1.399 (26,0)	866 (38,9)	1.012 (36,3)	1.885 (37,4)	648 (38,6)	788 (38,5)	1.436 (38,6)
≥12	127 (8,1)	126 (5,3)	253 (6,4)	239 (10,9)	265 (8,3)	504 (9,3)	410 (18,4)	419 (15,0)	835 (16,5)	435 (25,9)	418 (20,4)	853 (22,9)

a Pardo, amarelo e indígena.

**Figura 2:** Prevalência e intervalo de confiança 95% (IC95%) e evolução temporal de violência física e ataque físico em adolescentes escolares. Pernambuco, 2006, 2011, 2016 e 2022 (n=20.987)

**Tabela 2:** Razão de chances (*odds ratio*, OR) e intervalos de confiança de 95% (IC95%) ajustados da violência física pelas variáveis do estudo. Pernambuco, 2006-2022 (n=20.987)

Variáveis	2006			2011		2016		2022	
	OR (IC95%)	p-valor^a^	OR (IC95%)	p-valor^a^	OR (IC95%)	p-valor^a^	OR (IC95%)	p-valor^a^
Fatores individuais e familiares								
Sexo								
Feminino	1,00		1,00		1,00		1,00	
Masculino	1,10 (0,89; 1,37)	0,350	1,29 (1,05; 1,58)	0,014	1,21 (1,02; 1,44)	0,024	1,08 (0,87; 1,34)	0,453
Idade (anos)								
14	1,00		1,00		1,00		1,00	
15	0,91 (0,51; 1,62)	0,755	1,24 (0,70; 2,18)	0,451	0,95 (0,57; 1,59)	0,862	1,37 (0,57; 3,30)	0,474
16	1,07 (0,62; 1,85)	0,801	1,03 (0,59; 1,79)	0,906	0,90 (0,55; 1,50)	0,709	1,16 (0,48; 2,78)	0,727
17	0,84 (0,48; 1,45)	0,533	0,87 (0,50; 1,53)	0,649	1,00 (0,60; 1,65)	0,993	0,90 (0,37; 2,16)	0,817
18	1,05 (0,60; 1,83)	0,858	0,92 (0,51; 1,63)	0,776	0,80 (0,47; 1,36)	0,418	0,90 (0,37; 2,18)	0,815
19	0,77 (0,42; 1,41)	0,407	0,97 (0,52; 1,81)	0,934	0,92 (0,50; 1,71)	0,814	0,72 (0,26; 2,01)	0,539
Raça/cor da pele								
Branca	1,00		1,00		1,00		1,00	
Preto	1,12 (0,88; 1,43)	0,344	1,17 (0,91; 1,50)	0,210	1,09 (0,88; 1,35)	0,409	0,85 (0,67; 1,08)	0,208
Outra^b^	1,10 (0,66; 1,84)	0,695	1,68 (1,20; 2,36)	0,002	1,19 (0,88; 1,61)	0,237	1,23 (0,78; 1,92)	0,367
Escolaridade maternal								
Sem escolaridade	1,00		1,00		1,00		1,00	
1º grau completo	0,91 (0,68; 1,23)	0,577	0,98 (0,67; 1,44)	0,951	1,64 (1,01; 2,67)	0,044	0,77 (0,42; 1,39)	0,393
2º grau incompleto	1,09 (0,76; 1,57)	0,623	1,06 (0,69; 1,63)	0,763	1,85 (1,14; 3,00)	0,012	0,99 (0,54; 1,81)	0,990
2º grau completo ou mais	0,93 (0,66; 1,31)	0,703	0,87 (0,58; 1,30)	0,504	1,74 (1,09; 2,78)	0,020	0,92 (0,52; 1,64)	0,793
Fatores comportamentais								
Consumo de álcool								
Não	1,00		1,00		1,00		1,00	
Sim	1,60 (1,25; 2,00)	<0,001	1,34 (1,07; 1,67)	0,010	1,40 (1,16; 1,67)	<0,001	1,61 (1,27; 2,03)	<0,001
Consumo de tabaco								
Não	1,00		1,00		1,00		1,00	
Sim	1,47 (1,04; 2,07)	0,025	2,59 (1,84; 3,66)	<0,001	1,62 (1,22; 2,16)	0,001	1,57 (1,10; 2,24)	0,012
Consumo de drogas ilícitas								
Não	1,00		1,00		1,00		1,00	
Sim	2,18 (1,57; 3,03)	<0,001	2,08 (1,51; 2,86)	<0,001	1,88 (1,47; 2,40)	<0,001	2,56 (1,86; 3,52)	<0,001

a Regressão logística ajustada pelas variáveis inseridas na tabela; ^b^Pardo, amarelo e indígena.

**Tabela 3:** Razão de chances (*odds ratio*, OR) e intervalos de confiança de 95% (IC95%) ajustados dos ataques físicos pelas variáveis do estudo. Pernambuco, 2006-2022 (n=20,987)

Variáveis	2006			2011		2016		2022	
	OR (IC95%)	p-valor^a^	OR (IC95%)	p-valor^a^	OR (IC95%)	p-valor^a^	OR (IC95%)	p-valor^a^
Fatores individuais e familiares				
Sexo				
Feminino	1,00		1,00		1,00		1,00	
Masculino	1,44 (1,17; 1,77)	<0,001	2,33 (2,01; 2,71)	<0,001	2,17 (1,88; 2,51)	<0,001	1,24 (1,05; 1,47)	0,008
Idade (Anos)								
14	1,00		1,00		1,00		1,00	
15	2,76 (1,05; 7,29)	0,040	0,98 (0,65; 1,48)	0,956	1,02 (0,65; 1,59)	0,919	1,33 (0,69; 2,58)	0,391
16	3,04 (1,18; 7,85)	0,021	0,95 (0,64; 1,42)	0,832	0,87 (0,56; 1,35)	0,557	1,13 (0,59; 2,19)	0,697
17	3,96 (1,55; 10,1)	0,004	0,86 (0,58; 1,28)	0,462	0,96 (0,62; 1,48)	0,855	0,90 (0,46; 1,74)	0,766
18	4,73 (1,84; 12,1)	0,001	0,80 (0,53; 1,22)	0,311	0,80 (0,50; 1,26)	0,342	0,87 (0,44; 1,70)	0,688
19	5,07 (1,94; 13,2)	0,001	0,79 (0,50; 1,25)	0,327	0,51 (0,29; 0,89)	0,018	1,15 (0,54; 2,43)	0,703
Raça/cor da pele								
Branca	1,00		1,00		1,00		1,00	
Preto	1,10 (0,87; 1,41)	0,397	0,96 (0,81; 1,15)	0,704	0,95 (0,79; 1,13)	0,576	1,28 (1,05; 1,50)	0,012
Outra^b^	1,30 (0,80; 2,12)	0,280	1,22 (0,94; 1,57)	0,126	1,23 (0,96; 1,58)	0,101	1,95 (1,36; 2,77)	<0,001
Escolaridade maternal								
Sem escolaridade	1,00		1,00		1,00		1,00	
1º grau completo	0,92 (0,68; 1,23)	0,592	0,84 (0,64; 1,11)	0,240	1,36 (0,93; 1,99)	0,109	0,97(0,60; 1,56)	0,908
2º grau incompleto	0,79 (0,54; 1,16)	0,239	0,84 (0,61; 1,15)	0,296	1,64 (1,13; 2,39)	0,009	1,09 (0,67; 1,77)	0,710
2º grau completo ou mais	1,19 (0,80; 1,54)	0,496	0,92 (0,69; 1,23)	0,597	1,35 (0,94; 1,95)	0,099	1,14 (0,72; 1,81)	0,560
Fatores comportamentais								
Consumo de álcool								
Não	1,00		1,00		1,00		1,00	
Sim	4,26 (3,44; 5,28)	<0,001	1,81 (1,54; 2,13)	<0,001	2,02 (1,74; 2,36)	<0,001	1,91(1,59; 2,29)	<0,001
Consumo de tabaco								
Não	1,00		1,00		1,00		1,00	
Sim	1,56 (1,15; 2,12)	0,004	2,13 (1,60; 2,83)	<0,001	2,31 (1,80; 2,96)	<0,001	1,27 (0,93; 1,72)	0,121
Consumo de drogas ilícitas								
Não	1,00		1,00		1,00		1,00	
Sim	3,72 (2,77; 5,00)	<0,001	2,35 (1,84; 3,01)	<0,001	1,95 (1,57; 2,42)	<0,001	2,14 (1,63; 2,81)	<0,001

a Regressão logística; ^b^ Pardo, amarelo e indígena.

## Discussão 

Adolescentes que frequentam escolas públicas brasileiras possuem elevado percentual de violência e ataque físico, com prevalência maior no ano de 2016. Observou-se diminuição no sexo masculino e aumento no sexo feminino, de maneira que, no último ano avaliado, as prevalências de violência física ficaram semelhantes e próximas às de ataque físico. A exposição ao álcool e às drogas permaneceu como fator de risco em todos os anos avaliados. Contudo, o uso de tabaco não esteve associado a ataque físico no último ano de avaliação. O sexo masculino permaneceu mais exposto a agressões e raça/cor da pele aparecem como fator de risco.

Como limitação, este estudo não retratou a totalidade da população adolescente, especialmente aqueles que não frequentam a escola. Os resultados obtidos não devem ser generalizados para toda a população adolescente. Os vieses de resposta ou de memória e desejabilidade social podem diminuir o autorrelato de experiências de violência e comportamentos de risco à saúde. 

O aumento da prevalência de adolescentes vítimas de violências evidencia a necessidade de assistência constante e políticas públicas^19^. A violação dos direitos impacta diretamente ou indiretamente a saúde física e mental, além de influenciar seu rendimento escolar, relação social e familiar^20^. Observou-se associação de comportamentos e perfis, como: ser do sexo masculino, usar drogas lícitas e ilícitas, faltar às aulas, residir em áreas violentas, enfrentar conflitos familiares, ter supervisão parental insuficiente e depressão^21^^,^^22^.

A pesquisa *Health Behavior in School-aged Children* (HBSC) revelou que meninos têm maior probabilidade de se envolver em brigas do que meninas. Ademais, essa variação relacionada ao gênero foi notada em quase todos os países analisados^20^. Eventos violentos foram mais predominantes em instituições públicas e entre estudantes masculinos^12^.

Este estudo mostrou que tanto a violência quanto o ataque físico obtiveram diminuição no sexo masculino e aumento no feminino. Nesse sentido, consideram-se fatores relacionados à construção social, fundamentados na ideia de virilidade e força culturalmente enraizados na masculinidade, dinâmica de poder, banalização e aceitação da violência ^19^^,^^20^^,^^23^. Assim, há uma tendência de convergência nos comportamentos agressivos entre os gêneros, provavelmente afetada por elementos socioculturais, ambientais e tecnológicos ^24^.

A vulnerabilidade manifestada pela representação feminina é fruto de processos históricos e culturais marcados pelo machismo e patriarcado. No período de 2015 a 2016, mulheres lançaram a hashtag “#vamosjuntas?”, com o intuito de construir redes solidárias por meio das mídias sociais, para prevenir a violência em locais públicos e promover a colaboração entre mulheres, que possuem parcela maior do que o público masculino no acesso à internet^24^.

As plataformas virtuais são essenciais no cenário atual, permitindo a expansão, propagação e coordenação de ideias dos movimentos sociais. Além disso, buscam restaurar relações de poder, reestruturar interesses e fomentar a transformação social^25^. Com isso, torna-se necessário intensificar a implementação de políticas públicas e programas de prevenção da violência, no intuito de diminuir as disparidades sociais e fortalecer a legislação.

Observou-se que a exposição ao consumo de álcool, tabaco e substâncias ilícitas persistiu como fator de risco para a violência física. Em relação ao ataque físico, o sexo masculino, raça/cor da pele, o consumo de álcool e drogas ilícitas foram fatores de risco, e o consumo de tabaco deixou de apresentar-se como risco no último ano da coleta. Nessa vertente, nota-se que a prevalência do consumo de cigarro entre os adolescentes diminuiu. Porém, o consumo de álcool e outras drogas aumentou ao longo dos anos, e associa-se a comportamentos violentos^26^^,^^27^.

Esta pesquisa analisou os fatores preditores do consumo de álcool, de drogas ilícitas e da violência entre os adolescentes. No entanto, a complexidade em definir a ordem desses comportamentos tem dificultado a definição de relações causais entre eles^28^. Observou-se que o consumo de substâncias teve chance 2,4 vezes maior de estar associado à violência entre jovens ^29^. O consumo de álcool está associado à redução da capacidade de julgamento, do autocontrole e ao aumento de comportamentos de risco e violência^30^.

Nesse contexto, é essencial planejar e avaliar políticas públicas que visem prevenir a violência, promover a saúde e fomentar uma cultura de paz. Além disso, os profissionais precisam de capacitação para identificar situações de violência, além de integrarem as redes de assistência e suporte social.

## Data Availability

O banco de dados e os códigos de análise utilizados na pesquisa estão disponíveis para consulta, sob demanda justificada ao autor correspondente.
